# Diagnostic Accuracy of the Magnetic Resonance Imaging in Adult Post-Ganglionic Brachial Plexus Traumatic Injuries: A Systematic Review and Meta-Analysis

**DOI:** 10.3390/brainsci11020173

**Published:** 2021-01-30

**Authors:** Massimiliano Leigheb, Stefano Tricca, Ilaria Percivale, Davide Licandro, Andrea Paladini, Michela Barini, Giuseppe Guzzardi, Federico A. Grassi, Alessandro Stecco, Alessandro Carriero

**Affiliations:** 1 Orthopaedics and Traumatology Unit, “Maggiore della carità” Hospital, Department of Health Sciences, University of Piemonte Orientale (UPO), Via Solaroli 17, 28100 Novara, Italy; massimiliano.leigheb@uniupo.it (M.L.); federico.grassi@uniupo.it (F.A.G.); 2 Department of Diagnosis and Treatment Services, Radiodiagnostics, “Maggiore della carità” Hospital, University of Piemonte Orientale (UPO), Via Solaroli 17, 28100 Novara, Italy; stefano.tricca@libero.it (S.T.); 20004369@studenti.uniupo.it (D.L.); andreapaladini1988@gmail.com (A.P.); barinimichela@gmail.com (M.B.); giuseppe.guzzardi@maggioreosp.novara.it (G.G.); alessandro.stecco@uniupo.it (A.S.); profcarriero@virgilio.it (A.C.)

**Keywords:** brachial plexus, MRI scan, MRI diffusion weighted, nervous system traumas, peripheral nerves

## Abstract

Background: Traumatic brachial plexus injuries are rare but serious consequences of major traumas. Pre-ganglionic lesions are considered irreparable, while post-ganglionic injuries can be potentially treated if an early diagnosis is available. Pre-surgical diagnosis is important to distinguish low-grade from high-grade lesions and to identify their location. The aim of the review is to evaluate the diagnostic accuracy of magnetic resonance imaging (MRI) in the identification of adult post-ganglionic lesions due to traumatic brachial plexus injuries, compared to intraoperative findings. Methods: Research on the main scientific electronic databases was conducted. Studies of adults with traumatic post-ganglionic brachial plexus injuries were included. The index test was preoperative MRI and the reference standard was surgical exploration. Pooled sensitivity and specificity were calculated. Results: Four studies were included for the systematic review, of which three articles met the inclusion criteria for the meta-analysis. Pooled sensitivity and pooled specificity values resulted high. The sensitivity value is associated with a high heterogeneity index of the selected literature. Conclusion: MRI can be considered, despite the limits, the gold standard exam in morphological evaluation of brachial plexus injuries, particularly in the diagnosis of post-ganglionic traumatic injuries.

## 1. Introduction

The brachial plexus (BP) is the neural network that provides innervation to the upper chest, shoulders, and upper limbs. It is formed by the anterior branches of the last four cervical nerves (C5, C6, C7, and C8) and the first thoracic nerve (T1); the posterior and anterior nerve roots carry, respectively, sensory and motor fibers and exit from the spinal canal through the intervertebral foramen [1].

Before the union of the fibers there is an important structure, the posterior or dorsal root ganglion (DRG), which is considered an important landmark: lesions occurring proximally to DRG are defined pre-ganglionic, while lesions occurring distally to DRG are defined as post-ganglionic.

The second division of the BP is represented by three primary trunks: the superior trunk (formed by the union of C5 and C6 anterior roots), the middle trunk (which is the continuation of C7 anterior root), and the inferior trunk (C8 and T1 roots). The trunks are typically described as running into the interscalene triangle with the subclavian artery [2,3].

Near the lateral border of the first rib, each trunk splits into two branches: anterior and posterior. The six divisions form a triangular cluster that can be identified until the coracoid process occurs, where they form three cords.

The cords—lateral, posterior, and medial—run close to the axillary artery towards the pectoralis minor muscle, where they separate into five terminal branches: the axillary nerve, the median nerve, the musculocutaneous nerve, the radial nerve, and the ulnar nerve [1].

Traumatic BP injuries affect 1% of patients involved in major trauma (car accidents, occupational injuries, and falling), causing disability, pain, psychologic morbidity, and reduced quality of life [2,3,4].

According to the Seddon, Sunderland, and MacKinnon classifications, traumatic plexopathies can be divided into six degrees based on the number of layers damaged: neuropraxia (first degree), axonotmesis (from second to fourth degree), and neurotmesis (from fifth to sixth degree) [5,6].

Neuropraxia is a clinical condition characterized by temporary loss of function without denervation atrophy of the muscle. Axonotmesis is characterized by a Wallerian degeneration followed by nerve regeneration. While the latter can be managed conservatively, neurotmesis needs surgery for axon and myelin sheath disruption [7].

Another important classification of nerve injuries is based on their location: pre-ganglionic lesions are considered irreparable, while post-ganglionic injuries can be potentially surgically treated if an early diagnosis is available. Early surgical nerve repair leads to better functional recovery of the upper limb function [8,9].

As a consequence, diagnosis is important to distinguish low-grade lesions not requiring surgical treatment from high-grade lesions and to identify their location [10,11]. As magnetic resonance imaging (MRI) is a non-invasive, non-radiative imaging modality with multi-planar capability and great soft tissue characterization, it is a basic diagnostic imaging modality [12].

Many authors have examined the role of MRI in the diagnosis of traumatic BP injuries.

This review aims to evaluate the diagnostic accuracy of MRI in the identification of adult post-ganglionic lesions due to traumatic BP injuries, compared to intraoperative findings.

## 2. Materials and Methods

In this review, authors performed a systematic research of literature in order to perform a meta-analysis of studies reporting experience on traumatic BP injuries and evaluate the role of MRI in detecting these lesions. Database searching and study selection process were carried out from September 2020 to November 2020.

### 2.1. Eligibility Criteria

Inclusion criteria for this systematic review were

(1) studies focused on patients affected by post-traumatic post-ganglionic BP nerve injury with preoperative MRI;

(2) studies comparing MRI findings with surgical findings as gold standard;

(3) and meta-analysis studies reporting the number of true positive (TP), true negative (TN), false positive (FP), and false negative (FN).

Exclusion criteria were

(1) data concerning paediatric patients;

(2) studies utilizing animal models;

(3) studies considering only root avulsions or pre-ganglionic injuries;

(4) studies comparing MRI findings with only physical examination, CT myelography, or electrophysiological exams;

(5) and studies without clearly described MRI and surgical findings.

### 2.2. Database Search

A systematic research was conducted including the following electronic databases: Medline (via PubMed), Scopus (Elsevier), and Cochrane Central Register of Controlled Trials (CENTRAL). The detailed search strategies were first developed in Medline and then applied in the other databases. Selected articles from each database were at first screened for duplication.

Potentially relevant titles and abstracts found in the database search were stored, and a further detailed review of the full text was performed. There were no linguistic or year-of-publication limits to minimize the possibility of publication bias.

Qualified studies were selected according to the inclusion and exclusion criteria mentioned above.

Combined descriptors and keywords were selected for the search strategy in order to find the most relevant literature related to the topic, such as “brachial plexus”, “magnetic resonance imaging”, “nerve injuries”, “post-ganglionic”, “neuroma”, “accuracy”, “neuroimaging”, “diagnostic imaging”, “surgery”, “diagnosis”, “sensitivity”, “specificity”, and “predictive value”.

### 2.3. Study Selection

This study was conducted in accordance with the preferred reporting items for systematic reviews and meta-analyses–diagnostic test accuracy (PRISMA-DTA) statement [12].

Concerning the analysis, literature selection was conducted by two independent authors, and a third evaluator for detecting possible errors in selecting the articles. The results were then grouped and described in an Excel table. Extracted data included authors; year of publication; participant demographics; study design; index test; gold standard; and the number of TP, TN, FP, and FN subjects.

The quality of the included articles was assessed using the revised quality assessment of diagnostic accuracy studies (QUADAS-2) tool, through which the risk of bias was assessed in terms of quality of patient selection, index test, and reference standard [13]. In this study, the index test was MRI and the reference standard was surgery.

### 2.4. Statistical Analysis

Pooled sensitivity and specificity were calculated using Meta-DiSc, version 1.4.0, a Windows-based, freely available (for academic use) software that was developed, piloted, and validated to perform diagnostic meta-analysis. The same software was used to generate the forest plots [12]. These results were calculated using the number of TP, FN, FP, and TN subjects in all selected studies. Likelihood ratio (LR) evaluated the discriminatory properties of the test results. Positive and negative likelihood ratio evaluated the positive and negative test results, respectively. Pooled positive and negative likelihood ratio was calculated using a random effects model.

All final outcomes were presented with 95% confidential interval. Heterogeneity testing of the pooled results was assessed using the *I*^2^ statistic. When heterogeneity was significant (*I*^2^ > 75%), we considered exploring the sources of heterogeneity.

## 3. Results

### 3.1. Strategy Search

**Figure 1:**
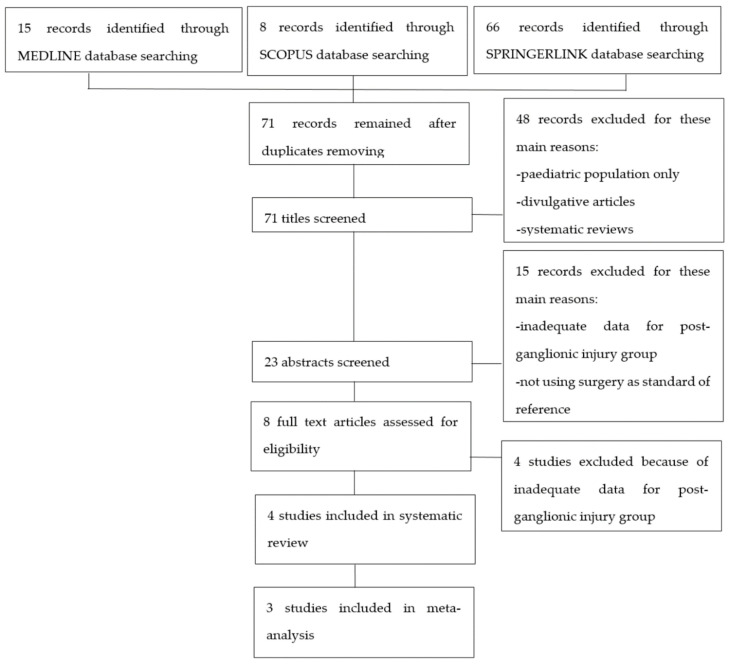
Flowchart of study selection process.

After searching in the aforementioned internet databases and removing duplicates, 71 articles were retrieved. These studies were then screened for eligibility as presented in the flow-chart (Figure 1). Eight articles underwent a full text screen and four of them were excluded because they were lacking adequate data regarding post-ganglionic BP injuries. Four studies were included in our systematic review, as summarized in Table 1. Of these, three were included in the meta-analysis [14,15,16,17], while Caporrino et al. [18] was excluded from the quantitative synthesis since TP, FP, TN, and FN were not reported in the text. All the included studies had prospective design and considered patients with traumatic BP injuries. All the studies but Caporrino et al. reported the number of patients included [15,16,17]. Two of the four studies provided information about the age range of the patients [16,17]. In Acharya, Caporrino, and Gad, a 1.5T MRI scanner was employed [15,16,18], while in Zhang, a 3T MRI scanner was used [17]. All the studies but Caporrino provided a precise description of the employed MRI protocol [15,16,17]. All the included studies used surgical findings as standard of reference [15,16,17,18].

### 3.2. Methodological Quality Assessment

Quality assessment of the included studies was conducted with the QUADAS-2 tool (Table 2) [11]. All the included studies but Zhang provided adequate information about patients’ inclusion and exclusion criteria [15,16,18]. MRI protocol was extensively described in all the studies but Caporrino [15,16,17]. It was mentioned in Gad only that surgeons were blinded to the MRI results, and therefore the reference standard was considered unlikely to have introduced biases [15]. Only Acharya’s article provided clear information about both the time intervals between injuries and MRI and between MRI and surgery; It was then considered at low risk of bias in terms of “flow and timing” [16]. Caporrino et al. only reported the time interval between injury and MRI [18].

### 3.3. Synthesis of Results

Table 1 shows characteristics and main conclusions of the selected studies.

MRI findings considered as significative for post-ganglionic injury of the BP were:

- nerve rupture: characterized by different degrees of nerve thickening caused by edema and inflammation with abnormal hyper intense signal in T2/short-tau inversion recovery (STIR) sequences;
- neuroma formation, characterized by a focal thickening of the injured segment of the nerve [17].

The selected studies did not clearly distinguish data among the different type of lesion.

Table 3 shows sensitivity, which refers to the true positive rate (true positives)/(true positive + false negative), and specificity, which refers to the true negative rate (true negatives)/(true negative + false positive), values with 95% confidence intervals of MRI for traumatic post-ganglionic lesions for each study included in the meta-analysis and the relative forest plots [15,16,17].

The paper written by Caporrino et al. was also selected for the systematic review and reported a sensitivity of 60% (95% CI 32.3–83.7) and a specificity of 59.8% (95% CI 48.7–70.1%) [18].

The pooled sensitivity, pooled specificity, and 95% confidence interval of the three studies included in the meta-analysis are shown in Table 4. Pooled sensitivity turned out to be of 90% (95% CI 0.78–0.97) and the pooled specificity of 90% (0.86–0.94). The sensitivity value is, however, associated with a *I*^2^ rate >75%, due to the heterogeneous results of the selected literature.

## 4. Discussion

Traumatic BP injuries may cause important disability, chronic pain, and consequent limitation in the activities of daily living. Although most lesions spontaneously heal, permanent limitations are not rare, especially in high-grade lesions [19,20].

The interest in assessing the best diagnostic strategy for BP traumatic lesions lies in the extreme importance of their early diagnosis and tempestive treatment. A delay in their identification, indeed, is related to an extremely poor prognosis [10,21,22].

The surgical treatment usually consists of a micro-reconstructive nerve surgery through direct nerve repair, nerve grafting, or nerve transfer.

In order to choose the most suitable therapeutic strategy, clinical examination alone is not enough as it is extremely challenging to differentiate pre-ganglionic from post-ganglionic injuries [1,23].

A correct diagnosis of brachial plexopathy generally involves both physical and instrumental examinations such as electromyography (EMG), nerve conduction studies, CT myelography, US, or MR imaging [24].

Nowadays, magnetic resonance is considered worldwide as the radiological gold standard for brachial plexopathy and peripheral nerve lesions [15,25].

MRI permits detailed investigation of peripheral nerve anatomy and pathology, as well as assessment of surrounding soft tissues and musculature, facilitating accurate diagnosis and preoperative planning [26,27].

Although many articles examine the role of MRI in the early diagnosis of traumatic BP injuries, only a few studies focus on the distinction between pre- and post-ganglionic lesions, particularly on the latter.

In this review, authors selected four studies [15,16,17,18].

In 2013, Caporrino et al. carried out a study aimed to determine the diagnostic performance of physical examination, of nerve conduction studies (NCS), and of MRI using surgery as reference standard in BP injuries. The sensitivity and specificity of the MRI in detecting post-ganglionic lesions were, respectively, 60% and 59.8%. The conclusion of this study was that, despite the poor performance of the single diagnostic strategies, NCSs and MRI used in conjunction with PE could increase the diagnostic accuracy [18]. The second study [17] focused on the role of MRI for detecting brachial plexopathies, reporting a sensitivity of 91.3%, a specificity of 60%, and an overall accuracy of 85.71% despite a small sample size. In 2019, Acharya et al. reported a sensitivity of 87% and a specificity of 26% of MRI, associated with a positive predictive value (PPV) of 26% and an negative predictive value (NPV) of 88% [16].

The most recent study [15] investigated the role of MRI in the diagnosis of adult and obstetric BP injuries and reported a sensitivity of 89%, a specificity of 100%, a PPV of 100%, and an NPV of 99% in detecting traumatic post-ganglionic adult injuries.

In this review, authors performed a meta-analysis of the last three articles. Although only a small number of studies were included, this paper can be considered a meta-analysis because it summarizes reported literature about traumatic BP injuries [4,28,29,30].

Our pooled sensitivity turned out to be 90% (95% CI 0.78–0.97) since in all the included studies values were comparably very high, showing the possibly important role of MRI in detecting traumatic BP lesions. On the other hand, the specificity values were very heterogeneous, indeed: our pooled specificity was 90% (0.86–0.94) with a *I*^2^ value of 98.1%. It is important to underline that Acharya et al. reported a very low specificity value, explained by the authors by a possible overestimation of the lesions in the presence of additional avulsion injuries that could have brought some changes distally. Because of the great heterogeneity of specificity data of the included studies, we believe that our pooled specificity value could be limited by some bias. As a consequence, if Gad et al. study was not included in the analysis, the sensibility obtained in the meta-analysis would not change (0.90, 95% CI 0.74–0.98) whereas the specificity would decrease significantly (0.31, 95% CI 0.61–0.50). Unfortunately, the poor number of studies in literature and reported in this paper affects the specificity value. It is important to underline that further studies are recommended in order to obtain more accurate values.

In conclusion, authors think that the association between imaging and clinical data could improve these parameters in any case, avoiding overestimation of post-ganglionic BP injuries, as stated by Caporrino et al. [18].

All MRI protocols were performed with 1.5T scanners, except for Zhang et al. [17], who used a 3T scanner. However, values of specificity and sensitivity in the last-mentioned study were not significantly higher, suggesting that the magnetic field is not relevant for the diagnosis of BP injuries. On the other hand, 1.5T scanner can offer a stronger fat saturation and an image quality less affected by susceptibility artifacts [27].

As far as imaging protocols are concerned, there was no significant difference among the studies: a standard protocol consists of a sagittal and a coronal T1 and T2 turbo spin-echo (TSE) or fast spin-echo (FSE) sequence, followed by a 3D STIR sequence.

Zhang and Gad also included a diffusion-weighted imaging with background signal suppression (DWIBS) on the coronal plane. This sequence was not performed by Achayra et al., and MRI protocol was not reported by Caporrino et al. [15,16,17,18].

**Figure 2:**
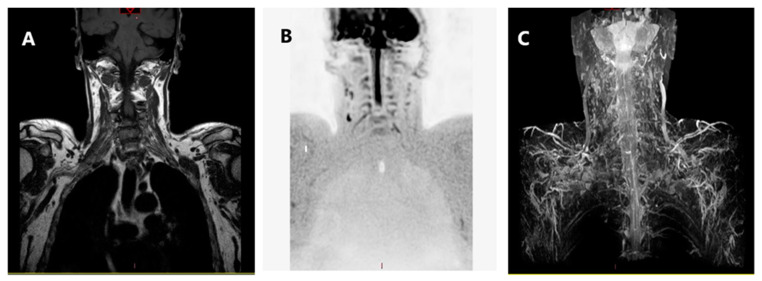
MRI sequences: (**A**) turbo spin-echo (TSE) T1 cor, (**B**) DWIBS, and (**C**) 3D STIR mip.

DWI sequence provides a better contrast differentiation between nerve fibers and surrounding tissues; our data may suggest a positive correlation between the use of DWIBS scan and values of specificity and sensitivity. Parametric data derived from these sequences may add quantitative information to BP study, increasing detection and characterization of neural damage and grading its severity [1,31] (Figure 2).

This is the first systematic review and meta-analysis focused on MRI diagnostic accuracy for post-ganglionic BP injuries, but there are some limitations.

First of all, there is very poor literature about the role of MRI on post-ganglionic BP injuries in adults. All the articles cited in this meta-analysis, indeed, have been published in the last five years.

Importantly, the selected studies show some heterogeneity in terms of timing of the MRI investigation. Acharya et al. performed MRI at least three weeks after injury [16], and Caporrino from two to three months after injury [18], while Gad and Zhang did not report the timing of the exam [15,17]. This is certainly a limitation of the current study and could have introduced a bias in studies comparison. Further studies are needed to identify any differences in MRI diagnostic performance as its timing after the injury varies, possibly indicating differences between the acute and the chronic setting.

In order to perform our meta-analysis, we only considered data regarding patients with post-ganglionic BP lesions. These cases represented a subgroup of the included studies, and no data regarding M/F proportion and mean age of such subgroups were available. For this reason, it was impossible to assess the homogeneity of the samples from which pooled data were extracted, and in this lies another important limitation of our work.

## 5. Conclusions

According to the current literature, MRI is extremely sensitive in detecting post-ganglionic BP traumatic lesions, of which it provides an excellent morphological characterization. The diagnostic accuracy could be improved and should be integrated with anamnesis, the physical examination, and electromyographic data.

Further studies are necessary to confirm literature current results.

## Figures and Tables

**Table 1:** Summary of included studies.

	Study Design	Subject Features	Postganglionic Lesions	Age	MRI Field Intensity	MRI Sequences Employed	MRI Timing	Standard of Reference	Level of Evidence	Main Conclusion
Acharya, 2019 [16]	Prospective	35 patients with traumatic brachial plexus injuries	Eight surgically demonstrated postganglionic lesions	Patients under the age of 60	1.5 T	T1-T2-T2 weighted 3D neurography-T2 spin echo- short-tau inversion recovery (STIR)	At least 3 weeks after injury	Surgery	2b	Magnetic resonance imaging (MRI) is a useful tool in the diagnosis of brachial plexus injuries.
Zhang, 2018 [17]	Prospective	28 patients with traumatic brachial plexus injuries	23 surgically demonstrated postganglionic lesions, in 12 patients	Mean age: 27.2	3 T	T1-T2-STIR- balance FFE- diffusion-weighted imaging with background signal suppression (DWIBS)	Not reported	Surgery	2b	MRI is a valuable diagnostic tool for brachial plexus lesions, especially if balance-FFE, STIR, and DWIBS sequences are performed.
Caporrino, 2014 [18]	Prospective	34 patients with traumatic plexus injuries	Not reported	Mean age: 29.8	1.5 T	Not reported	2–3 months after injury	Surgery	2b	MRI showed poor diagnostic performance in identifying brachial plexus lesions compared to physical examination. Notwithstanding, it is reasonable to think that the combination of physical examination and MRI could provide the best diagnostic accuracy.
Gad, 2020 [15]	Prospective	22 patients with traumatic brachial plexus injuries	18 surgically demonstrated postganglionic lesions	Mean age: 26.3	1.5 T	T1, STIR, T2, T2-STIR, and DWIBS	Not reported	Surgery	2b	“MRI is the imaging modality of choice in the examination of traumatic and obstetric brachial plexus injuries; it is safe and non-invasive, having the multiplanar capability and better soft tissue characterization”.

**Table 2:** Quality assessment of diagnostic accuracy studies (QUADAS)-2, quality assessment of the included studies.

	Patient Selection	Index Test	Reference Standard	Flow and Timing
Acharya, 2019 [16]	+	+	+	+
Zhang, 2018 [17]	?	+	?	?
Caporrino, 2014 [18]	+	?	+	?
Gad, 2020 [15]	+	+	+	?

**Table 3:** Forest plot showing sensitivity and specificity for each included study.

Study	TP	FP	FN	TN	Sensitivity (95% CI)	Specificity (95% CI)	Forrest Plots
Gad 2020 [15]	16	0	2	198	0.89 (0.65–0.99)	1.00 (0.98–1.00)	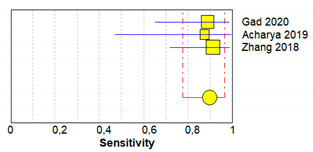 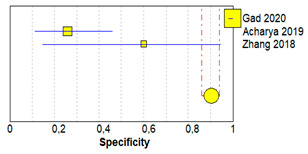
Acharya 2019 [16]	7	20	1	7	0.88 (0.47–1.00)	0.26 (0.11–0.46)	
Zhang 2018 [17]	21	2	2	3	0.91 (0.72–0.99)	0.60 (0.15–0.95)	

Abbreviations: TP, true positive; FP, false positive; FN, false negative; TN, true negative.

**Table 4:** Results of pooled data.

TP	FP	FN	TN	Pooled Sensitivity		Pooled Specificity		Pooled LR+		Pooled LR−		Pooled DOR	
				Value (95% CI)	*I* ^2^	Value (95% CI)	*I* ^2^	Value (95% CI)	*I* ^2^	Value (95% CI)	*I* ^2^	Value (95%CI)	*I* ^2^
44	22	5	208	0.90 (0.78–0.97)	0.0%	0.90 (0.86–0.94)	98.1%	7.70 (0.28–214.76)	96.5%	0.17 (0.07–0.39)	0.0%	40.71 (0.99–1666.3)	84.6%

Abbreviations: TP, true positive; FP, false positive; FN, false negative; TN, true negative; CI, confidence interval; LR+, positive likelihood ratio; LR−, negative likelihood ratio; DOR, diagnostic odd ratio.
